# A retrospective study about incidental appendectomy during the laparoscopic treatment of intussusception

**DOI:** 10.3389/fped.2022.966839

**Published:** 2022-09-06

**Authors:** Tao Liu, Yibo Wu, Weijue Xu, Jiangbin Liu, Qingfeng Sheng, Zhibao Lv

**Affiliations:** Department of General Surgery, Shanghai Children's Hospital, School of Medicine, Shanghai Jiao Tong University, Shanghai, China

**Keywords:** intussusception, laparoscopy, incidental appendectomy, child, surgery

## Abstract

**Purpose:**

We aim to see incidental appendectomy (IA) was worth or not during the laparoscopic treatment of intussusception.

**Methods:**

This study included forty-eight patients who underwent a laparoscopic procedure for idiopathic intussusception without intestinal resection between April 2014 and April 2021. The Chi-square or Fisher's exact tests for categorical variables and the Student *t*-test for continuous variables were used to analyze and compare patient characteristics.

**Results:**

IA was performed on 63% (30/48) of patients after surgical reduction, while 18 (37%), did not. Patients who underwent IA had a higher total cost (16,618 ± 2,174 vs.14,301 ± 5,206, *P* = 0.036), and a longer mean operation duration (59 ± 19 vs.45 ± 21, *P* = 0.025). The distribution of the PO time, length of hospital stay, PCs, and RI did not differ significantly. The histopathological evaluation of the 30 resected appendices revealed five (17%) with signs of acute inflammation, 20 (66%) with chronic signs of inflammation, and five (17%) with inconspicuous appendices.

**Conclusion:**

IA is linked to a longer average operation time and a higher total cost. There is insufficient evidence to recommend IA during laparoscopic intussusception treatment. The risks and benefits of IA need further study.

## Introduction

Intussusception is the prolapse of one section of the intestine into the lumen of the adjacent distal part, which is a common cause of intestinal obstruction in young children (1). The specific types can be described as idiopathic intussusception which has no pathologic leading point (PLP), and secondary intussusception with PLP (2). When classified by anatomic types according to the start of the intussusceptum and the end of the intussuscipiens, generally, it can be divided into different types: ileocolic, colocolic, jejunojejunal, ileoileal, ileoileocolic, and so on (1, 2). The treatment plan is determined by the clinical manifestations of the patients. Contrast enema reduction should be conducted on patients with stable hemodynamics. Otherwise, surgery will be required (3–5), and minimally invasive techniques may be used to avoid laparotomy (3, 5).

IA refers to the removal of the vermiform appendix accompanying another operation, without evidence of acute appendicitis (6). It is debatable whether IA should be performed during intussusception surgery. IA was done to eliminate the risk of future appendicitis (6), and to avoid future confusion if the patient developed lower abdominal pain at a later age, especially in the female patient (7, 8). Adhesive bands depending on the extent of scar tissue from the original operation may complicate a second operation (6). In addition, proponents of IA point to the ease of resection, low morbidity, lack of additional anesthetic risks, and high disease incidence in specimens (9). However, Bonnard et al. didn't support IA because their study showed that the reserve of the appendix did not increase the risk of recurrent intussusception (10). Others suggest that IA should be reconsidered in light of medical advances such as enhanced imaging techniques and the use of the appendix as a tubular conduit for reconstruction (6, 11), and the development of laparoscopy, which has fewer complications including intra-abdominal adhesion formation compared with open appendectomy (6, 12). Besides, IA may increase the infectious risk for elevating the case from a “clean” to a “clean-contaminated” operation. So, we conducted this research in the hopes of resolving this conundrum.

## Materials and methods

Ethical approval was obtained from the Ethics Boards of Shanghai Children's Hospital. We reviewed the medical records of 540 patients who were diagnosed with intussusception and admitted to Shanghai Children's Hospital between April 2014 and April 2021. Among them, forty-eight patients with idiopathic intussusception treated with laparoscopic reduction satisfied our inclusion criteria. Patients who needed a gut resection as part of their operational care (because of intestinal necrosis or PLP) were excluded from the study.

Patients' symptoms include paroxysmal sobbing, vomiting, a jam-like blood stool, or an abdominal mass, and confirmed by B ultrasound or air enema. For patients with no contraindications, air enema reduction is the first option at our facility. Patients who have failed to reduce air enema, have unstable hemodynamics, or were in a serious condition were treated with emergency surgery. All surgical procedures were carried out by surgeons with at least 5 years of experience in our department. IA could be performed based on the surgeon's preference and the intraoperative macroscopy of the appendix at the time of surgery. Patients with a red and swollen appendix were more likely to get IA than those with a normal appendix. The procedure for laparoscopic reduction is as follows: atraumatic graspers were utilized to locate the distal intussusceptum, two atraumatic graspers were alternately squeezed to reduce the intussusceptum, and combined by pulling the proximal small intestine. The region is then thoroughly examined to confirm a seamless transition along the serosa and to ensure a successful reduction. And any PLPs, such as polyps or Mickel's diverticulum, should be thoroughly checked. The surgeon performs a laparoscopic appendectomy using a common procedure. A LigaSure is used to clamp and control the mesoappendix. An Endoloop is placed at the base of the appendix. The resected appendix was then taken out of the abdomen using a laparoscopic retrieval bag. The patients were discharged with no symptoms and were able to resume their normal diet and feces.

Gender, age, duration of symptoms, times of air enemas before surgery, operative time, anatomic types of intussusception, perioperative complications (PCs), time to oral intake after the operation (PO time), recurrent intussusception (RI), length of intussusceptum, intraoperative macroscopy of the appendix, length of hospital stay, total cost, histopathological evaluation of the appendix, adhesive small bowel obstruction (adhesive SBO), appendicitis and so on were among the information collected. Participants were classified into two groups: the study group, representing those requesting an AI after reduction, and the control group, referring to those not requesting an appendectomy. Follow-up was performed by telephone or review data of the outpatient system.

Descriptive statistics were reported as absolute frequencies and percentages for qualitative variables or medians and ranges for continuous variables. The Student's *t*-test was used to analyze continuous variables. The Chi-square and Fisher's exact tests were used when appropriate to study categorical variables. All statistical analyses were performed using Stata version 20.0 (StataCorp LP, College Station, TX, USA). Analysis items with a *P*-value of < 0.05 were considered statistically significant.

## Results

Table 1 shows the background characteristics of 48 patients (34 men and 14 females) who were reviewed. They were 16 months old on average (range, 5–107months). It took an average of 20 h from the onset of symptoms until the diagnosis (range, 5–96 h). Ninety percent of the children had at least one air enema, with the majority having two. Ileocolic intussusception was the most common form of intussusception, with forty cases, followed by small bowel in five cases, ileoileocolic in two cases, and ileocolocolic in one. Three patients (6%) experienced PCs, with one having serosal torn during surgery and a fever thereafter, one having serosal torn alone, and one having an intestinal infection. None of those patients had wound infections. The mean follow-up time was 51 months (range, 22–95 months), and the last follow-up time was on 3 August 2022. All patients' information was got. Only four patients (8%) had RI, and the time interval for RI was at least 12 months. A fresh intussusception following a successful non-surgical or surgical reduction is referred to as a recurrent intussusception. Ultrasound was used to confirm all recurrences. None of those patients occurred with adhesive SBO after surgery during the follow-up time.

**Table 1:** Characteristics of patients.

**Items**	**Results**	**Items**	**Results**
Gender (cases)	Male (34)	With or without IA	
	Female (14)	Without IA	18 (37%)
Age (months)	Scale (5–107)	With IA	30 (63%)
	Median (16)	With or without PCs	
Duration of symptoms (h)^*^	Scale (5–96)	Without pcs	45 (94%)
	Mean (20)	With pcs	3 (6%)
Times of air enemas		Serosal torn	1
		and fever	
0	5 (10%)	Serosal torn	1
1	7 (15%)	Infection	1
2	29 (60%)	Follow time (months)	scale (22–95)
			mean (51)
3	7 (15%)	With or without RI	
Operation time (min)	Scale (23–115) Mean (54)	Without RI	44 (92%)
Types of intussusceptin		With RI	4 (8%)
Ileocolic	40 (83%)	Time to RI (months)	scale (12–24)
Small bowl	5 (11%)		mean (16)
Ileoileocolic	2 (4%)	Appendicitis	0
Ileocolocolic	1 (2%)	Adhesive SBO	0
PO time (h)	Scale (8–112)		
	Mean (36)		

* Excluding one case of a chronic small intussusception, whose duration of symptoms is longer than 20 days.

IA, incidental appendectomy. PCs, perioperative complications. PO time, time to oral intake after the operation. RI, recurrent intussusception. Adhesive SBO, adhesive small bowel obstruction.

**Figure 1:**
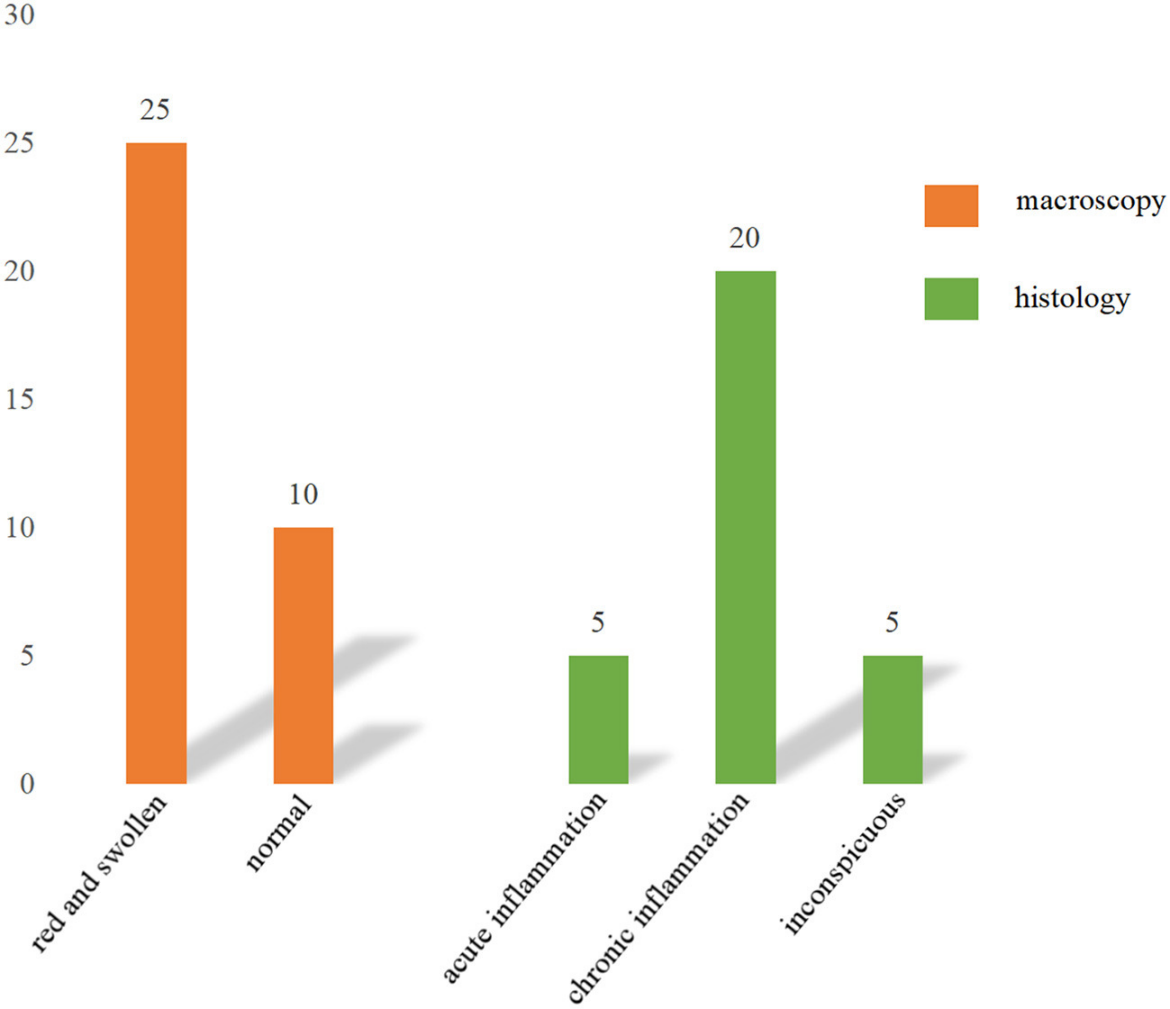
Intraoperative macroscopy and histology of the appendixes.

IA was done on 63% (30/48) of patients, whereas 18 (37%) did not. None of the patients with their appendix reserved developed appendicitis during our follow-up. Table 2 shows the comparison between patients with and without IA. Patients with IA had a higher total cost (16,618 ± 2174 vs. 14,301 ± 5,206, *P* = 0.036), and had a longer mean operation duration (59 ± 19 vs. 45 ± 21, *P* = 0.025). The distribution of the PO time, length of hospital stay, PCs, and RI, however, did not differ significantly. The histological examination of the 30 removed appendices revealed 5 (17%) with evidence of acute inflammation, 20 (66%) with chronic signs of inflammation, and 5 (17%) with inconspicuous appendices (Figure 1).

**Table 2:** The comparison between patients with and without IA.

**Items**	**IA**	**No IA**	***P***
Operation time (min)	59 ± 19	45 ± 21	0.025
PO time (h)	33.6 ± 20.5	42.3 ± 21.0	0.220
Length of hospital stay (days)	6.0 ± 1.2	6.2 ± 2.7	0.808
Total cost (yuan)	16,618 ± 2,174	14,301 ± 5,206	0.036
With or without PCs (cases)			
Without	28 (93.3%)	17 (94.4%)	0.687
With	2 (6.7%)	1 (5.6%)	
With or without RI (cases)			
Without	28 (93.3%)	16 (88.9%)	0.484
With	2 (6.7%)	2 (11.1%)	

Surgeons decide whether to proceed with an IA or not depending on their preference and the intraoperative macroscopy of the appendix. Factors that may influence surgeons' decisions are shown in Table 3. Patients who underwent IA had more preoperative air enemas (2.0+0.7 vs. 3+0.8, *P* = 0.004). The distribution of the kind of intussusception, length of intussusceptum, and duration of symptoms did not differ significantly.

**Table 3:** The factors which may influence surgeons' decision.

**Items**	**IA**	**No IA**	***P***
Times of air enemas	2.0 ± 0.7	1.3 ± 0.8	0.004
Types of intussusception (cases)			
Ileocolic	26 (86.7%)	14 (77.8%)	0.082
Small bowl	1 (3.3%)	4 (22.2%)	
Ileoileocolic	2 (6.7%)	0 (0.0%)	
Ileocolocolic	1 (3.3%)	0 (0.0%)	
Intraoperative macroscopy of the			
appendixes (cases)			
Red and swollen	23 (88.5%)	2 (22.3%)	0.001
Normal	3 (11.5%)	7 (77.8%)	
Length of intussusceptum (cm)	8.3 ± 5.5	9.1 ± 7.6	0.726
Duration of symptoms (h)	17.3 ± 10.8	25.5 ± 20.8	0.081

## Discussion

For almost a century, experts have discussed the risks and benefits of IA during intussusception procedures in children. The incidental procedure is to prevent, given the patient's age and gender; effectiveness in preventing the associated disease; additional risk and complications secondary to the incidental procedure; and cost of the incidental procedure concerning the cost of treating the disease in those affected, as Sugimoto and Edwards stated (13). As for children, the future utility of the appendix shouldn't be neglected (6). So, it's hard to reach a consensus.

Our findings reveal that patients with IA had a substantially higher overall cost (16,618 ± 2,174 vs. 14,301 ± 5,206, P=0.036). For patients with appendix reserved, none of them occurred with appendicitis during our follow-up. So far in our study, they have benefited financially. But in the long run, it's hard to determine whether or not it is a cost-effective procedure, due to a lack of relevant data. According to Albright et al. (14) there is a cost-benefit for men <55 years old with benign colon and rectal illness, with savings ranging from $8,131 per 10,000 population in the 0–5 year age group to $725 per 10,000 population in the 50–54 year age group. For women under the age of 50, there is also a cost advantage, particularly for those in the 0–5 year age range. Those patients, on the other hand, received open procedures. Due to additional expenses such as LigaSure, Endoloop, and Laparoscopic retrieval bag, the cost of laparoscopy must be re-addressed. According to Wang and Sax, if IA was conducted during open surgery, a cost-benefit may be obtained for people under the age of 25, but there was no cost-benefit in any age group if the treatment was performed laparoscopically due to extra equipment (15).

Another advantage for patients with their appendix left in place is that they have shorter surgery time (59 ± 19 vs. 45 ± 21, *P* = 0.025). An IA took an average of 14 min to perform. According to Albright et al. (14) the expected time to do a laparoscopic IA is 10 min, which is less than ours. While others reported 12.3 min (8). This may be because we're pediatric surgeons with less experience than adult surgeons. As we all know, the longer the procedure takes, the more anesthesia is used, which not only adds to the expense but also adds to the anxiety of the parents.

Besides, the appendix has become more recognized as a structure of considerable utility for reconstructive surgeries as medical science has progressed, such as urological reconstruction, colonic irrigation, and biliary reconstruction. The appendix's potential applications are increasingly being addressed while deciding whether or not to proceed with IA (6).

The reserve of the appendix, on the other hand, may raise the risk of appendicitis, recurrent intussusception, and other rare appendix disorders in children, including torsion of the appendix, a strangulated internal hernia through an appendicular ring or through a mesoappendix gap, an incarcerated appendix in an acute hernia sac, and appendiceal intussusception in the future (16). Appendiceal intussusception is a rare disease in children characterized by varying degrees of appendix invagination into the cecum (17), and a few case reports have been published (18–20). However, recurrent intussusception has a variety of causes (21). Some surgeons recommend removing the appendix, which might act as a PLP (22). While a PLP from a bowel suture line or appendiceal stump was also described as the cause of an exceedingly uncommon intussusception following an IA (23). In our investigation, there was no significant difference in the rate of recurrent intussusception between the two groups. And none of the patients with their appendix reserved developed appendicitis during our follow-up.

As for patients with IA, a small part of them may benefit from this process when the histological analysis of the removed appendix showed clinically significant founding, such as neoplasms. But this phenomenon is closely associated with patients' age. In our research, histological analysis revealed five inconspicuous appendices (17%). The acute-inflammatory transformation was found in five specimens (17%). Twenty specimens (66%) exhibited indications of chronic inflammation. Although some patients showed a histologic anomaly, it was usually small and did not lead to a new diagnosis. The outcome is similar to that of a younger adult (31.1 ± 11.3 years) who had IA during a living donor hepatectomy (24). While in order patients (62.9 ± 13.9 years), pathologic findings of clinical significance in the appendix, such as carcinoid tumors and gastrointestinal stromal tumors, were present in 2.6% of specimens (24). Another study, including patients with a mean age of 61 years, reported neoplasms were found in 3.8% of the resected appendixes in an IA surgery (25).

One disadvantage of an IA is that it elevates the case from a “clean” to a “clean-contaminated” operation, posing an increased infectious risk, according to the guidelines for antimicrobial prophylaxis in surgery (26). But laparoscopy in a patient with no risk factors can lower the infection risk rate from 1.31 to 0.67 percent (6). In our study, all patients underwent a laparoscopic procedure, and none of them developed an incision infection, whether they had IA or not. Only three patients (6%) had perioperative complications, including one who had a serosal tore during surgery and had a fever after, one had a serosal tore, and another one had an intestinal infection. There was no statistical difference in wound infections or surgical complications between the two groups of patients, which was consistent with the findings of A. Wang et al. (7). Al-Temimi et al. found 743 (0.37%) IA among 199,233 abdominal operations and discovered that IA was associated with higher wound complications (OR = 1.46, 95% CI = 1.05–2.03), but their patients included adults who had undergone open surgery (27). Another disadvantage of IA is that it's potentially contributing to the development of gastrointestinal malignancies, as new research suggests that the appendix may also act as a reservoir for the colonic microbiome, suggesting that removing the appendix may alter the components of the gastrointestinal microbiome (28).

The intraoperative macroscopy of the appendix differs between them, which might be because the patient who had IA had more preoperative air enemas (2.0 ± 0.7 vs. 1.3 ± 0.8, *P* = 0.004), resulting in a red and swollen appendix. Patients with a red and large appendix were more likely to undergo IA, but those with a normal appendix were not, potentially confounding our findings. There are further restrictions. Other variables influencing the surgeon's choice, such as palpable fecalith within the appendix vermiformis, should be taken into account. Second, while PO time and hospital stay are dependent on the patient's health and the surgeon's choice, a consistent treatment plan should be used. Furthermore, our retrospective research was done at a single institution with a limited sample size. Multicenter, prospective research with a larger study population is required to address the aforesaid issues.

## Conclusion

There is insufficient evidence to recommend IA during laparoscopic intussusception treatment. The appendix's intraoperative macroscopy may affect surgeons' decisions. So, more research is required.

## Data availability statement

The original contributions presented in the study are included in the article/supplementary material, further inquiries can be directed to the corresponding author.

## Ethics statement

Ethical approval was obtained from the Ethics Boards of Shanghai Children's Hospital. Written informed consent to participate was not required per local legislation and institutional requirements.

## Author contributions

TL and QS had the idea for the article. TL performed the data acquisition, data analysis, data interpretation, and manuscript preparation. ZL, WX, JL, and YW contributed to the critical review of the intellectual content of this manuscript. All authors contributed to the article and approved the submitted version.

## Conflict of interest

The authors declare that the research was conducted in the absence of any commercial or financial relationships that could be construed as a potential conflict of interest.

## Publisher's note

All claims expressed in this article are solely those of the authors and do not necessarily represent those of their affiliated organizations, or those of the publisher, the editors and the reviewers. Any product that may be evaluated in this article, or claim that may be made by its manufacturer, is not guaranteed or endorsed by the publisher.
